# A hand hygiene intervention to reduce infections in child daycare: a randomized controlled trial

**DOI:** 10.1017/S095026881400329X

**Published:** 2015-01-08

**Authors:** T. P. ZOMER, V. ERASMUS, C. W. LOOMAN, A. TJON-A-TSIEN, E. F. VAN BEECK, J. M. DE GRAAF, A. H. E. VAN BEECK, J. H. RICHARDUS, H. A. C. M. VOETEN

**Affiliations:** 1 Department of Infectious Disease Control, Municipal Public Health Service Rotterdam-Rijnmond, Rotterdam, The Netherlands; 2 Department of Public Health, Erasmus MC, University Medical Centre Rotterdam, Rotterdam, The Netherlands

**Keywords:** Cold (common), gastroenteritis, hand hygiene, hygiene – professional, infectious disease control

## Abstract

Infections are common in children attending daycare centres (DCCs). We evaluated the effect of a hand hygiene (HH) intervention for caregivers on the incidence of gastrointestinal and respiratory infections in children. The intervention was evaluated in a two-arm cluster randomized controlled trial. Thirty-six DCCs received the intervention including HH products, training sessions, and posters/stickers. Thirty-five control DCCs continued usual practice. Incidence of episodes of diarrhoea and the common cold in children was monitored by parents during 6 months. Using multilevel Poisson regression, incidence rate ratios (IRRs) with 95% confidence intervals (CIs) were obtained. Diarrhoeal incidence was monitored in 545 children for 91 937 days. During follow-up, the incidence was 3·0 episodes per child-year in intervention DCCs *vs.* 3·4 in control DCCs (IRR 0·90, 95% CI 0·73–1·11). Incidence of the common cold was monitored in 541 children for 91 373 days. During follow-up, the incidence was 8·2 episodes per child-year in intervention DCCs *vs.* 7·4 in control DCCs (IRR 1·07, 95% CI 0·97–1·19). In this study, no evidence for an effect of the intervention was demonstrated on the incidence of episodes of diarrhoea and the common cold.

## INTRODUCTION

Children attending daycare centres (DCCs) acquire gastrointestinal and respiratory infections more often than children cared for at home [1, 2]. Hand hygiene (HH) is considered to be a simple and effective measure to prevent infections [3, 4]. However, in DCCs caregivers' compliance with HH guidelines is low [5].

Although several HH interventions have been developed to reduce DCC-related infections [6–14], inconsistent results on their effectiveness have been reported [15]. Moreover, these interventions were not reported as being developed according to a stepwise behavioural approach using models and theories from the behavioural sciences to understand the determinants that underlie HH behaviour [16]. Our previous research showed that environmental determinants, such as the availability of paper towels, are associated with caregivers' HH compliance in DCCs [5]. In addition, we found that the following sociocognitive determinants are associated with HH compliance of DCC caregivers: knowledge and awareness of HH guidelines, perceived importance of performing HH, perceived behavioural control (i.e. perceived ease or difficulty of performing the behaviour), and habit [17]. Interventions aiming to improve caregivers' HH compliance in DCCs are more likely to be successful when addressing these determinants. We therefore developed an intervention based on these sociocognitive and environmental determinants of caregivers' HH behaviour. The aim of the intervention was to increase caregivers' compliance with HH guidelines (primary outcome measure) and reduce infections in children (secondary outcome measure). Due to the intervention, caregivers' compliance with HH guidelines improved. Compliance was defined as the number of HH actions divided by the total number of opportunities for which HH was indicated. According to the Dutch national guidelines, HH is mandatory for caregivers before touching/preparing food, before caregivers themselves ate or assisted children with eating, and before wound care; and after diapering, after toilet use/wiping buttocks, after caregivers themselves coughed/sneezed/wiped their own nose, after contact with body fluids (e.g. saliva, vomit, urine, blood, or mucus when wiping children's noses), after wound care, and after hands were visibly soiled [18]. HH compliance was observed at 1, 3 and 6 months follow-up. At 6 months follow-up, caregivers' HH compliance in intervention DCCs was 59% *vs.* 44% in control DCCs (baseline-corrected OR 4·13, 95% CI 2·33–7·32) (T. P. Zomer *et al*., unpublished data). The effect of our intervention on HH compliance will be described in a forthcoming paper. In this paper we assess the effect of our intervention on incidence of gastrointestinal and respiratory infections in children attending DCCs.

## METHODS

A cluster randomized controlled trial of a HH intervention was performed in DCCs in the regions of Rotterdam-Rijnmond, Gouda and Leiden in The Netherlands between September 2011 and April 2012. DCCs were randomized, stratified for size and urbanicity [19]. DCCs which participated in our previous study on HH determinants [5, 17], were contacted to participate in the trial.

The intervention consisted of four components [19]. First, the following HH products were provided free of charge: dispensers for paper towels, soap, alcohol-based hand sanitizer and hand cream, with refills for 6 months. Second, training about the Dutch national HH guidelines was given and a booklet outlining the content of the training was distributed. Third, two team training sessions were given aimed at goal-setting and formulating specific HH improvement activities. The team training sessions were based on similar HH training sessions developed for Dutch hospitals [20, 21]. Fourth, posters and stickers for both caregivers and children were provided as reminders and cues to action. Two groups in each DCC participated in the study. In intervention DCCs, these groups received the HH products. As caregivers rotated between groups, all caregivers received the training sessions. The intervention was implemented in four phases (HH products at the start, three training sessions with a 1-month interval). Intervention DCCs were compared to control DCCs which continued their usual practice.

The outcome measure was incidence of gastrointestinal and respiratory infections in children monitored by parents. Parents were enrolled in the trial between 1 August 2011 and 1 November 2011. Baseline measurement was collected between mid-September 2011 until 1 November 2011; starting when parents were enrolled and ending when the intervention started. Follow-up measurement was from 1 November 2011 until the end of March 2012. Children were recruited from two groups of the DCC, even if the DCC had more than two groups in total. In that case, in both intervention and control DCCs the researchers in collaboration with the managers of the DCCs randomly selected two groups. Parents were recruited from 142 (48%) groups out of a total of 297 groups. Children were eligible to participate if they: attended the DCC at least 2 days a week; were aged between 6 months and 3·5 years at start of the trial; intended to attend the DCC throughout the study period; and if their parents consented, were Dutch speaking, and had access to email or regular post. Children were excluded if they had a chronic illness or medication that predisposed them to infection, a sibling taking part in the trial (i.e. one child per family could be included), or if they started attending the DCC after start of the trial.

Parents were asked to monitor disease incidence in their child using an infection calendar to mark the days their child had diarrhoea and/or a common cold. Diarrhoea was defined as at least two watery or unusually loose stools in 24 h. The common cold was defined as a blocked or runny nose with at least one of the following symptoms: coughing, sneezing, fever, sore throat, or earache. Every 2 weeks, parents were contacted by email and regular post to enter the calendar page in an online version of the calendar or to send it in using a free-of-charge return envelope. Parents who did not respond were reminded after 1 week (email), 2 weeks (letter), and 3 weeks (telephone). Sample size calculation showed that to be able to detect 25% reduction in incidence of gastrointestinal infections of three per year and 15% reduction in incidence of respiratory infections of nine per year, we would need disease monitoring of 600 children for 6 months (80% power, two-sided alpha of 0·05) [19].

In order to interpret results we assessed exposure to the intervention. We observed whether the intervention dispensers and posters/stickers were in use at 6 months' follow-up. In addition, a survey was conducted among caregivers.

Data were analysed using SPSS version 19 (SPSS Inc., USA) and R version 2.12.2 (R Foundation, Austria). Analyses were performed according to the intention-to-treat principle, i.e. including all intervention DCCs irrespective of whether they used the HH products, posters/stickers or completed all training sessions. First, baseline characteristics were compared using *χ*^2^ test or Fisher's exact test for categorical variables and independent *t* test for continuous variables. Second, the incidence of diarrhoea and the common cold was calculated during baseline and follow-up in intervention and control DCCs. Incidence was defined as the number of disease episodes per child-year. A new disease episode was defined after seven symptom-free days and in additional analyses after three symptom-free days [9]. Episodes of illness which started on the first day parents started monitoring disease incidence were excluded.

Multilevel Poisson regression analyses were performed to correct for clustering of the data within DCCs. Incidence risk ratios (IRRs) with 95% confidence intervals (CIs) were obtained for the intervention effect, corrected for DCC group type (0–1, 2–3, 0–4 years), as this was the only possible confounder that was shown to be significantly different between intervention and control DCCs/children at baseline. Besides overall incidence, incidence was calculated stratified for children aged 0–1 and 2–3 years. Tests for overdispersion were performed, but no corrections were necessary.

Additional analyses were performed to correct for baseline measurement. For this we calculated the interaction between intervention status of the DCC (i.e. intervention *vs.* control) and follow-up measurement (i.e. baseline *vs.* follow-up). This resulted in an IRR for the difference between baseline and follow-up measurement in intervention DCCs and an IRR for the difference between baseline and follow-up in control DCCs. Comparison of these two IRRs resulted in a baseline-corrected IRR.

Ethical approval was waived by the Medical Ethics Committee of the Erasmus University Medical Centre in Rotterdam (MEC-2011–256).

## RESULTS

**Fig. 1.**
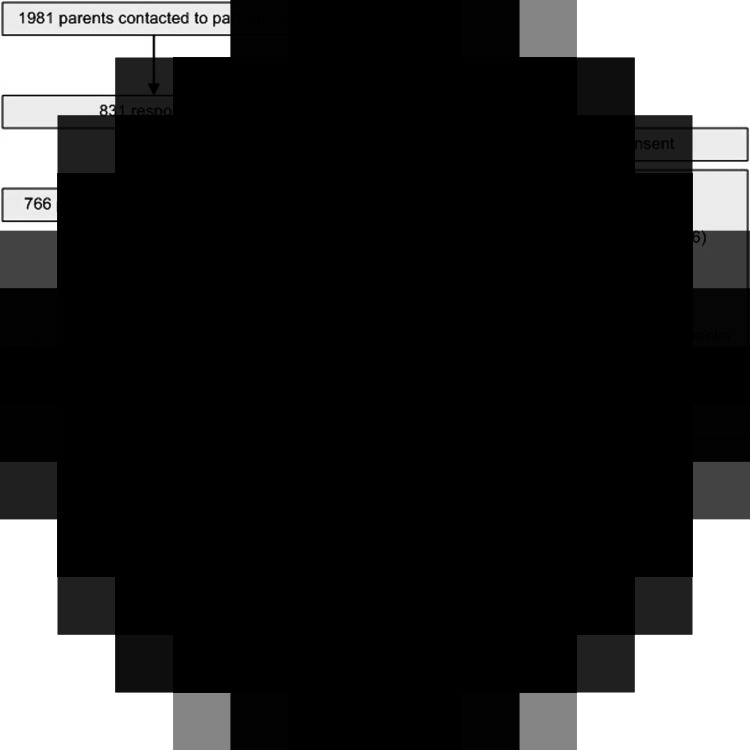
Flow diagram of the recruitment of children in 71 child daycare centres (DCCs).

In the trial 71 DCCs participated. After randomization, there were 36 intervention DCCs and 35 control DCCs. Of 1981 parents invited to participate, 766 gave informed consent for their child (response rate 39%) (Fig. 1). Of 766 children, 553 were eligible for inclusion. Of 553 children, five parents did not return any of the calendar pages with incidence data and three parents did not return any pages during follow-up, therefore 545 children were included in the analyses. For 19 of 545 children, baseline incidence data were missing.

Of the 545 children, 278 (51%) were in 34 intervention DCCs and 267 (49%) in 35 control DCCs. The median number of participating children per DCC was seven (range 1–18). Of the 545 parents, 94% returned ⩾12 of 14 calendar pages. Comparison of baseline characteristics demonstrated that the group type significantly differed between intervention and control DCCs (Table 1). This variable was therefore included in further analyses as a possible confounder. None of the other baseline characteristics was significantly different between intervention and control DCCs and children (Table 1).

**Table 1.** Comparison of baseline characteristics of intervention and control daycare centres (DCCs) and children

	Intervention DCCs	Control DCCs	*P* value
DCC characteristics	(*N* = 36)	(*N* = 35)	
Size (large, having ⩾46 children per day)	53%	51%	0·91
Degree of urbanicity			0·84
Highly urban	58%	63%	
Urban	22%	23%	
Slightly/non-urban	19%	14%	
Region			0·47
Rotterdam-Rijnmond	67%	66%	
Gouda	14%	6%	
Leiden	19%	29%	
Hygiene and quality certification (certified)	44%	41%	0·83
	Intervention children	Control children	
Child characteristics	(*N* = 278)	(*N* = 267)	
Gender (boys)	51%	54%	0·43
Age at start of trial (mean)	1·5 years	1·6 years	0·53
Children eating solid foods	98%	97%	0·74
Children solely breastfed at start of trial	0%	2%	0·056
Children ever breastfed	76%	79%	0·32
Number of days per week at the DCC (mean)	2·7 days	2·7 days	0·70
Children with siblings	56%	63%	0·09
Children with siblings at the DCC	25%	26%	0·65
Children that started attending the DCC in the 3 months before trial start	3%	3%	0·96
Children in a single-parent household	7%	9%	0·43
DCC group type			<0·001
0–1 years	16%	18%	
2–3 years	14%	32%	
0–4 years	70%	50%	

### Intervention exposure

All 36 intervention DDCs received the training on HH guidelines and all, but two, received at least one of the team training sessions. Another two intervention DCCs did not use any of the provided HH products. The response rate to the questionnaire on intervention exposure was 50% (274/546). Of 274 caregivers, 79% attended at least one of the training sessions. The information booklet on HH guidelines was received by 77% of caregivers. At 6 months follow-up, the dispensers for paper towels, soap, alcohol-based hand sanitizer, and hand cream were used in at least one of two groups in respectively 94%, 89%, 86%, and 45% of intervention DCCs. Moreover, in 86% the posters were used and in 74% the stickers.

### Incidence of episodes of diarrhoea and the common cold

**Fig. 2.**
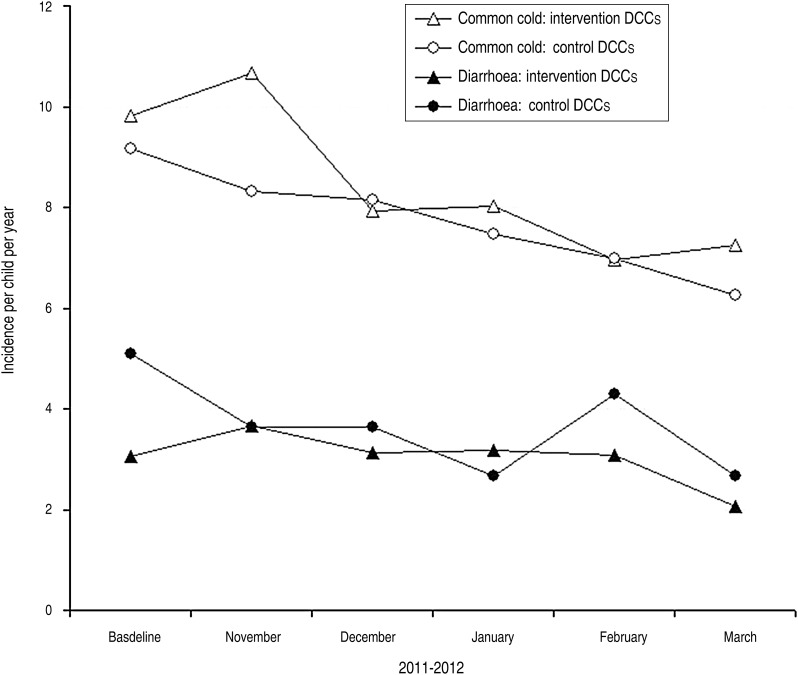
Effect of a hand hygiene intervention on incidence of episodes of diarrhoea and the common cold in children attending daycare centres (DCCs).

Incidence of episodes of diarrhoea was monitored in 545 children during 91 937 days. Incidence of episodes of the common cold was monitored in 541 children during 91 373 days. Of 545 children, four children were excluded from analyses because they had the common cold every day during the trial. Figure 2 shows the incidence of episodes of diarrhoea and the common cold in intervention and control DCCs over time; the crude incidence of diarrhoeal episodes differed between intervention and control DCCs at baseline, while during follow-up it was similar. Concerning the crude incidence of episodes of the common cold, at baseline as well as follow-up this was similar for intervention and control DCCs (except for November).

When defining a new episode of diarrhoea after seven symptom-free days, the incidence in intervention DCCs at baseline was 3·0 diarrhoeal episodes per child-year *vs.* 5·1 in control DCCs (Table 2). Corrected for group type and clustering of the data within DCCs, this difference was statistically significant (IRR 0·57, 95% CI 0·38–0·85). During follow-up there were 3·0 diarrhoeal episodes per child-year in intervention DCCs *vs.* 3·4 in control DCCs (IRR 0·90, 95% CI 0·73–1·11). The baseline-corrected IRR was 1·58 (95% CI 1·05–2·36). Additional analyses stratified by age, showed similar results during follow-up for children aged 0–1 year (IRR 0·97, 95% CI 0·75–1·26; baseline-corrected IRR 1·82, 95% CI 1·08–3·08) and children aged 2–3 years old (IRR 0·83, 95% CI 0·63–1·09; baseline-corrected IRR 1·29, 95% CI 0·68–2·43) (results not shown in table). Analyses with a new disease episode defined after three symptom-free days, showed that uncorrected for baseline incidence there were slightly fewer episodes of diarrhoea in intervention *vs.* control DCCs (IRR 0·81, 95% CI 0·63–1·05, *P* = 0·07) (Table 2).

**Table 2.** Effect of a hand hygiene intervention on incidence of episodes of diarrhoea in children attending daycare centres (DDCs) (N = 545)

	Intervention DCCs		Control DCCs		IRR* (95% CI)		*P* value
7 symptom-free days between episodes							
Baseline incidence†	3·0	(42/5 042)	5·1	(100/7 170)	0·57	(0·38–0·85)	0·006
Follow-up incidence†	3·0	(336/40 564)	3·4	(361/39 161)	0·90	(0·73–1·11)	0·32
IRR* (95% CI)	1·06	(0·76–1·48)	0·67	(0·54–0·84)	1·58	(1·05–2·36)	0·03
3 symptom-free days between episodes							
Baseline incidence†	3·2	(44/5 042)	5·7	(112/7 170)	0·53	(0·34–0·83)	0·002
Follow-up incidence†	3·3	(370/40 564)	4·1	(435/39 161)	0·81	(0·63–1·05)	0·07
IRR* (95% CI)	1·11	(0·77–1·60)	0·72	(0·57–0·92)	1·53	(1·00–2·36)	0·03

IRR, Incidence risk ratio; CI, confidence interval.

* Also corrected for clustering of the data within DCCs and group type.

† Incidence of episodes per year (i.e. no. of episodes/no. of days at risk).

When defining a new episode of the common cold after seven symptom-free days, the incidence in intervention DCCs at baseline was 9·8 episodes of the common cold per child-year *vs.* 9·2 in control DCCs (IRR 1·06, 95% CI 0·85–1·34) (Table 3). During follow-up there were 8·2 episodes of the common cold in intervention DCCs *vs.* 7·4 in control DCCs (IRR 1·07, 95% CI 0·97–1·19; baseline-corrected IRR 1·01, 95% CI 0·79–1·29). Additional analyses stratified by age, showed similar results during follow-up for children aged 0–1 year (IRR 1·07, 95% CI 0·93–1·22; baseline-corrected IRR 1·13, 95% CI 0·80–1·61) and children aged 2–3 years (IRR 1·10, 95% CI 0·95–1·27; baseline-corrected IRR 0·90, 95% CI 0·63–1·28) (results not shown in table). Analyses with a new disease episode defined after three symptom-free days, showed similar results as analyses with a new disease episode after seven symptom-free days (IRR 1·04, 95% CI 0·95–1·13; baseline-corrected IRR 1·05, 95% CI 0·84–1·33) (Table 3).

**Table 3.** Effect of a hand hygiene intervention on incidence of episodes of the common cold in children attending daycare centres (DDCs) (N = 541)

	Intervention DCCs		Control DCCs		IRR* (95% CI)		*P* value
7 symptom-free days between episodes							
Baseline incidence†	9·8	(132/4 914)	9·2	(178/7 096)	1·06	(0·85–1·34)	0·60
Follow-up incidence†	8·2	(904/40 354)	7·4	(794/39 009)	1·07	(0·97–1·19)	0·15
IRR* (95% CI)	0·83	(0·69–1·00)	0·82	(0·70–0·97)	1·01	(0·79–1·29)	0·94
3 symptom-free days between episodes							
Baseline incidence†	11·1	(194/4 914)	11·1	(216/7 096)	0·98	(0·80–1·21)	0·87
Follow-up incidence†	9·5	(1 048/40 354)	8·9	(955/39 009)	1·04	(0·95–1·13)	0·44
IRR* (95% CI)	0·86	(0·72–1·02)	0·81	(0·70–0·94)	1·05	(0·84–1·33)	0·65

IRR, Incidence risk ratio; CI, confidence interval.

* Also corrected for clustering of the data within DCCs and group type.

† Incidence of episodes per year (i.e. no. of episodes/no. of days at risk).

## DISCUSSION

This is the first HH intervention in DCCs developed according to a stepwise behavioural approach targeting the underlying determinants of caregivers' compliance with HH guidelines. The study objective was to evaluate the effect of the intervention on the incidence of episodes of diarrhoea and the common cold in children attending DCCs. During follow-up, there were fewer episodes of diarrhoea in intervention DCCs *vs.* control DCCs. However, this difference was not statistically significant and corrected for baseline the effect changed direction, with significantly more diarrhoeal episodes in intervention DCCs. This was primarily influenced by an unexplainable high baseline incidence in control DCCs. No effect of the intervention was shown on the incidence of episodes of the common cold.

Our study has several strengths. This is the first HH intervention in DCCs which was developed based on the underlying determinants of HH behaviour. In addition, this is one of few DCC intervention studies correcting for baseline incidence in multilevel analyses. Other strengths of the study are the randomized controlled design, a large sample size of 71 DCCs, high exposure to intervention components, and a high percentage of calendar pages returned by parents with few children lost to follow-up. In addition, control DCCs received the intervention after data collection, which probably facilitated DCC recruitment and also minimized loss to follow-up [7].

Our study has several limitations. First, as the response of parents was initially limited, there were not enough children included when starting baseline measurement. Therefore, recruitment of parents continued during baseline measurement. As a result, the number of days that parents completed the infection calendar during baseline varies. For four children, no baseline data were provided by the parents. Moreover, the exact weeks that baseline data were collected vary between the children, which might lead to incomparable results due to different circulating pathogens. An additional complicating factor is that half of intervention DCCs, due to practical reasons, had already received the training on HH guidelines while baseline measurement was still ongoing. For children of these DCCs, we censored the calendar days from the day of the training until the official intervention start on 1 November 2011. As a result, more baseline data were collected in control DCCs *vs.* intervention DCCs. The above-mentioned limitations of the baseline measurement might partly explain the baseline difference between intervention and control DCCs in incidence of episodes of diarrhoea. Another limitation is that our study is under-powered. According to sample size calculations, we would have needed disease monitoring of 600 children for 6 months (109 200 child-days) [19]. We monitored 545 children during 5 months follow-up, resulting in data on 79 725 child-days, which is 73% of the anticipated 109 200 child-days. Furthermore, the possible effect size of the intervention is probably smaller than what we assumed during sample size calculation. Other limitations are that the method to assess disease incidence was not validated, and the relatively low response rate in parents of 39%. No information was obtained on parents unwilling to participate. It might be that these parents have less interest in hygiene which could have influenced disease incidence at child DCCs and possibly also the intervention effect.

In the same trial we also assessed caregivers' HH compliance at baseline and follow-up, and found that HH compliance increased significantly in intervention *vs.* control DCCs (T. P. Zomer *et al*., unpublished data). At baseline, compliance in intervention DCCs was 53% *vs.* 63% in control DCCs (OR 0·59, 95% CI 0·37–0·94). At 6 months follow-up, compliance was 59% *vs.* 44%, respectively (baseline-corrected OR 4·13, 95% CI 2·33–7·32). Nevertheless, we were unable to demonstrate an effect of the intervention on incidence of episodes of diarrhoea and the common cold. This might partly be explained by the fact that on average the children attended the DCCs 2·7 days a week and children can also become infected outside the DCC. In The Netherlands it is common that at least one of the parents works part-time and therefore it is not surprising that the children only attend the DCC part-time. Another explanation could be that within DCCs other hygiene activities are also important for the prevention of disease transmission (i.e. cleaning toys, floors, furniture, toilets, etc.). Furthermore, in our study the main focus was on caregivers' HH; besides posters and stickers for children, there were no other techniques to encourage children's HH, even though their HH might also be important to reduce infections in DCCs. Another possible explanation is that HH compliance did not improve enough in intervention DCCs to result in a reduction in infections, or that the difference in HH compliance between intervention and control DCCs was not large enough to detect differences in disease incidence. There is possibly a critical threshold for HH compliance to result in a lower incidence of infections. A Dutch study on DCC-related disease burden, during the same time period as our intervention showed a peak in incidence of gastroenteritis in February 2012 [22]. In our control DCCs there was the same increase in incidence, while this was not the case in intervention DCCs. Therefore, it might be that with an increase of infections, the intervention becomes more effective.

Our study shows the importance of baseline measurements in intervention studies, as baseline incidence of diarrhoeal episodes differed between intervention and control DCCs. There are few other DCC intervention studies which performed a baseline measurement [6–8, 13]. One of these studies performed analyses to assess whether the difference between baseline and follow-up was different for intervention *vs.* control DCCs [8]. As we found a significant difference between intervention and control DCCs in diarrhoeal baseline incidence, we corrected the effect of the intervention for baseline incidence. This was done by adding an interaction term to assess whether the difference between baseline and follow-up was different for intervention *vs.* control DCCs. By adding this interaction term, we were still able to also correct for group type and clustering of the data within DCCs. To our knowledge, this is the first study to perform this type of analysis. More DCC intervention studies are needed with baseline measurement.

Previous HH intervention studies in DCCs have shown varying effects on incidence of gastrointestinal and/or respiratory infections and/or illness absenteeism [15]. We found three randomized controlled trials with an outcome measure of incidence of gastrointestinal and/or respiratory infections that corrected for clustering of the data [6, 9, 10, 13]. Gudnason *et al.* also reported a baseline measurement and, similar to our study, did not demonstrate an effect of their intervention on incidence of diarrhoea and colds [6]. Roberts and colleagues reported a reduction in episodes of colds only in children aged ⩽24 months and a reduction in episodes of diarrhoea only in children aged >24 months [9, 10]. However, in that study no baseline incidence was reported. Therefore, it is possible that the difference between intervention and control DCCs was already present before start of the intervention. Carabin *et al.* report that their intervention reduced the incidence of upper respiratory tract infections [13]. However, similar to our study, they also report a reduction in incidence in control DCCs. Therefore, it is less likely that the incidence reduction in intervention DCCs is caused by the intervention. This indicates that there is limited evidence available that improved HH in DCCs is associated with fewer gastrointestinal and respiratory infections. More evidence is needed to understand the importance of HH in reducing gastrointestinal and respiratory infections in children attending DCCs.

In conclusion, this study shows that there is no evidence that our HH intervention – addressing determinants that underlie caregivers' HH behaviour – is effective in reducing gastrointestinal and respiratory infections in children attending DCCs. An explanation might be that HH compliance did not increase enough to result in fewer infections and/or that other transmission routes are also important, such as other hygiene/cleaning activities within the DCC as well as children's HH. Future intervention studies should target several transmission routes and be evaluated in robust studies including baseline measurement.
